# Diverse distribution patterns of segmental longitudinal strain are associated with different clinical features and outcomes in dilated cardiomyopathy

**DOI:** 10.1007/s12574-024-00646-y

**Published:** 2024-03-07

**Authors:** Kaoruko Sengoku, Tomohito Ohtani, Yasuharu Takeda, Toshinari Onishi, Fusako Sera, Misato Chimura, Shozo Konishi, Yasuhiro Ichibori, Masayoshi Yamamoto, Tomoko Ishizu, Yoshihiro Seo, Yasushi Sakata

**Affiliations:** 1 Department of Cardiovascular Medicine, Osaka University Graduate School of Medicine, 2-2 Yamadaoka, Suita, 565-0871 Japan; 2 Cardiovascular Center, Sakurabashi-Watanabe Hospital, Osaka, Japan; 3 Department of Cardiology, Osaka Police Hospital, Osaka, Japan; 4 Laboratory Medicine, Faculty of Medicine, University of Tsukuba, Tsukuba, Japan; 5 Department of Cardiology, Nagoya City University Graduate School of Medical Sciences, Nagoya, Japan

**Keywords:** Longitudinal strain, Dilated cardiomyopathy, Prognosis, Reverse remodeling

## Abstract

**Background:**

Dilated cardiomyopathy (DCM) presents with diverse clinical courses, hardly predictable solely by the left ventricular (LV) ejection fraction (EF). Longitudinal strain (LS) offers distinct information from LVEF and exhibits various distribution patterns. This study aimed to evaluate the clinical significance of LS distribution patterns in DCM.

**Methods:**

We studied 139 patients with DCM (LVEF ≤ 35%) who were admitted for heart failure (HF). LS distribution was assessed using a bull’s eye map and the relative apical LS index (RapLSI), calculated by dividing apical LS by the sum of basal and mid-LS values. We evaluated the associations of LS distribution with cardiac events (cardiac death, LV assist device implantation, or HF hospitalization) and LV reverse remodeling (LVRR), as indicated by subsequent LVEF changes.

**Results:**

Twenty six (19%) and 29 (21%) patients exhibited a pattern of relatively apical impaired or preserved LS (defined by RapLSI < 0.25 or > 0.75, signifying a 50% decrease or increase in apical LS compared to other segments), and the remaining patients exhibited a scattered/homogeneously impaired LS pattern. The proportion of new-onset heart failure and LVEF differed between the three groups. During the median 595-day follow-up, patients with relatively-impaired apical LS had a higher rate of cardiac events (both log-rank *p* < 0.05) and a lower incidence of LVRR (both *p* < 0.01) compared to patients with other patterns. RapLSI was significantly associated with cardiac event rates after adjusting for age, sex, and new-onset HF or global LS.

**Conclusion:**

DCM patients with reduced EF and distinct distribution patterns of impaired LS experienced different outcomes.

**Supplementary Information:**

The online version contains supplementary material available at 10.1007/s12574-024-00646-y.

## Introduction

Dilated cardiomyopathy (DCM) is one of the major etiologies of heart failure (HF) with reduced left ventricular (LV) ejection fraction (EF). It has various clinical courses, including slow progression with asymptomatic LV dysfunction, the recovery of the LV function called LV reverse remodeling (LVRR), or the development of advanced HF due to irreversible severe LV dysfunction [1–4]. However, predicting the aforementioned outcomes using the indices obtained in clinical imaging is still challenging. Longitudinal strain (LS) has been developed to compensate for the weakness of EF in clinical settings [5–7]. One of the strengths of LS is its ability to detect subtle differences in systolic dysfunction among patients with preserved and reduced EF [6–9]. Global longitudinal strain (GLS) is a reliable predictor of prognosis or LVRR in patients with reduced EF [9–11]. Notwithstanding, diverse clinical courses in patients with similar GLS values still exist [9–11].

LS also reflects the regional abnormality of the myocardium, characterized by an apical preserved pattern. Furthermore, it helps differentiate cardiac amyloidosis from patients with preserved EF and hypertrophied heart [12]. However, reports regarding LV heterogeneous abnormalities in patients with non-ischemic cardiomyopathy and reduced EF are limited except in tachycardia-induced cardiomyopathy [13]. In addition, its clinical significance in DCM has not yet been elucidated. DCM is characterized by a dilated and generalized hypokinetic LV motion. However, the morphology of the left ventricle, such as its diameter, is diverse and accompanied by differences in the degree of hypokinetic regional wall motion. Therefore, we hypothesized that there could be some distribution patterns of impaired LS in DCM patients with reduced EF. These patterns will facilitate the precise evaluation of clinical features and outcomes in these patients.

## Methods

### Study subjects

We retrospectively screened 217 patients (185 at Osaka University Hospital and 32 at the University of Tsukuba Hospital) with DCM and LVEF ≤ 35%, without a history of open-heart surgery, including mitral valve repair and LV reduction surgery, or ischemic heart disease. These patients were hospitalized for HF at either hospital from January 2009 to December 2017. DCM was diagnosed according to the World Health Organization/International Society and Federation of Cardiology criteria [14] as cardiomyopathy with LV dilatation and a reduced EF without ischemic heart disease, valvular heart disease, or secondary cardiomyopathy by several examinations, including coronary angiography and endomyocardial biopsy. Cardiac amyloidosis was ruled out in all patients by endocardial biopsy or clinical examinations including scintigraphy, wall thickness assessments, and laboratory data. We excluded 44 patients because of poor images for acquiring the speckle tracking data. Furthermore, 34 patients were excluded due to a scheduled hospitalization to implant a left ventricular assist device (LVAD). Therefore, 139 patients were included in this study. This study complies with the principles of the Declaration of Helsinki. The study protocol was approved by the ethics committees of each hospital (17371-4 and H30-053).

### Echocardiographic assessments

Transthoracic echocardiographic data including LS were collected in stable conditions after admission. Echocardiography was performed by experienced sonographers and doctors using a commercially available ultrasound machine (EPIQ and iE33, Philips Healthcare, Andover, MA, USA; Vivid7 and Vivid E9, GE Healthcare, Milwaukee, WI, USA; Aplio Artida, Canon Medical Systems, Otawara, Japan). We obtained the measurements and recordings based on the American Society of Echocardiography recommendations [15]. The LVEF and left atrial volume index were calculated using the biplane disk summation method with two-dimensional images and were indexed to the body surface area. Based on a previous report, we calculated the wall stress on the left ventricle at diastole and systole [16]. The height and body weight were also obtained on echocardiography. In addition, we calculated the body mass index.

### Two-dimensional strain echocardiography

Peak systolic LS measurements were obtained from the gray-scale images, recorded in the apical four- and two-chambers and long-axis views. The frame rate was maintained at > 50 frames/s. After gathering all DICOM data at Osaka University Hospital, we analyzed the LV strain offline using the speckle tracking software (TOMTEC, TomTec Imaging Systems GmbH, Munich, Germany). Good image quality was defined as a clear detection of the endocardial border throughout the cardiac cycle, and the regions of interest at the apex and annulus were ensured. Following the manual definition of the LV endocardial border, the endocardium was automatically tracked throughout the cardiac cycle. The software algorithm automatically divided the LV apical view into six segments for the speckle tracking throughout the cardiac cycle. We obtained the GLS by averaging all the segmental LS values from the aforementioned chambers and long-axis views. LS values for the basal, mid, and apical LV segments (six each) were averaged to obtain the regional LS values (basal, mid, and apical, respectively). The relative apical LS index (RapLSI) is one of the indices used for evaluating the regional abnormality of the myocardium. It is calculated by dividing the apical LS by the sum of the basal and mid-LS values [12]. In this study, we divided the patients into three groups according to RapLSI. When apical LS was more than 50% higher (relatively-impaired) or lower (relatively preserved) than the average of LS in mid and basal segments, RapLSI becomes < 0.25 (Apical relatively-impaired group) or > 0.75 (Apical relatively preserved group). The patients with RapLSI from 0.25 to 0.75 were grouped as a Scattered/Homogenously impaired group. The inter- and intra-observer intraclass correlation coefficients for obtaining the RapLSI were determined for a subset of 20 patients. The estimated inter- and intra-observer intraclass correlation coefficients were 0.931 [0.811–0.974] and 0.975 [0.938–0.990], respectively.

### Clinical and follow-up data

We obtained data on the patient characteristics and laboratory tests, including the brain natriuretic peptide (BNP) levels on admission. HF duration was defined as the duration from the initial event that requires hospitalization for decompensated HF to indexed hospitalization by asking the patient and/or searching hospital records. Medication data were collected at the discharge of indexed hospitalization, and optimal medical therapy (OMT) scores were calculated according to the previous report [17]. Details on all cardiac deaths, LVAD implantation, and HF-associated re-hospitalization after the discharge of indexed hospitalization were collected from the medical records, and the first event was used for survival analyses. One year after discharge, data on LVEF and beta-blocker use (carvedilol equivalent dose) were collected to assess LVRR. LVRR was defined as achieving ≥ 10% unit increase in the LVEF and ≥ 10% decrease in the LV end-diastolic dimension [18]. At the follow-up, the patients on LVAD support were classified as having non-reverse remodeling.

### Statistical analyses

Continuous data are expressed as mean and standard deviation for normally distributed variables and as median and interquartile range for non‐normally. We compared the categorical variables using the Chi-square (*χ*^2^) test. The continuous variables were compared using a one-way analysis of variance with Bonferroni correction or the Steel–Dwass test for multiple unadjusted comparisons, and when appropriate, following an assessment of the normal distribution. Cochran–Mantel–Haensze test or two-way ANOVA was used to adjust for differences between institutions, as appropriate. The correlations among echocardiographic and clinical parameters were evaluated by the Pearson correlation coefficient. We conducted the Kaplan–Meier method to test for differences in the event-free rate between groups using the log-rank test. We used the Cox proportional-hazards model to adjust for the effects of differences in the baseline characteristics or pertinent covariates on the outcomes. Statistical comparisons were performed using the JMP, version 13 (SAS Institute Inc., North Carolina). All analyses were two-sided, and the significance was set at *p* < 0.05.

## Results

### Patient characteristics

**Fig. 1:**
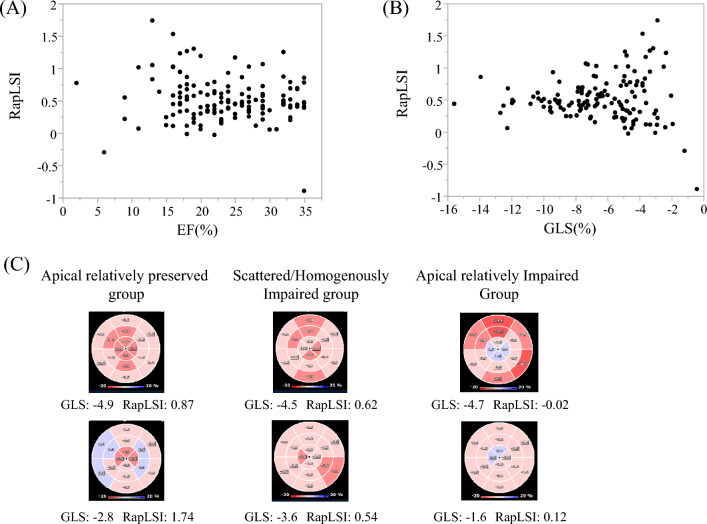
**A** Relationship between the RapLSI and EF. There is no significant correlation. **B** Relationship between the RapLSI and GLS. There is no significant correlation. However, the RapLSI has considerably dispersed in the lower part of the GLS. **C** The typical LS bull’s eye map of the apical relatively preserved, scattered/homogenously impaired, and apical relatively-impaired groups in severely decreased GLS. GLS, global longitudinal strain; LS, longitudinal strain; RapLSI, relative apical longitudinal strain index; and LVEF, left ventricular ejection fraction

Tables 1 and 2 summarize the differences in patient characteristics and echocardiographic data among the three groups of impaired LS patterns. Through the groups, reduced LVEF and enlarged LV end-diastolic dimension were consistent with DCM characteristics. The GLS value was low. The RapLSI was not correlated with EF and GLS (Fig. 1A, B). The RapLSI was widely distributed, particularly among the low GLS values. Consequently, GLS in the scattered/homogeneously impaired group differed from that in the other groups. Figure 1C shows the representative LS patterns of the three groups. In the patients in the apical relatively preserved group, new-onset HF was more often, but LVEF was more reduced. In contrast, patients in the apical relatively-impaired group tended to have more RV-paced rhythms compared with the other groups. No significant differences were observed in atrial fibrillation, medication, left and RV diastolic dimensions, or both systolic and diastolic wall stress on the left ventricle among the groups. Although there were some differences in clinical data between the two institutions (Supplemental Table 1 and 2), the differences in clinical characteristics among the three LS distribution groups after adjustment for the examined institution were consistent with those before adjustment (Tables 1, 2). In evaluating the associations between LS and HF duration, we observed a poor but significant correlation of HF duration with that of an apical LS (*p* = 0.02, *r* = 0.20) but not with the basal (*p* = 0.17) and mid-LS (*p* = 0.26). More impaired GLS in all patients tended to be correlated with longer HF duration (*p* = 0.07). In a limited number of patients with a scattered/homogeneous pattern, a weak association between the GLS and HF duration was observed (*p* < 0.01, *r* = 0.32).

**Table 1:** Patient characteristics

	Apical relatively-impaired group (*n* = 26)	Scattered/homogeneously impaired group (*n* = 84)	Apical relatively-preserved group (*n* = 29)	*p* value	Adjsuted *p* value
Age, years	61 [50–70]	54 [43–66]	49 [35–62]	0.12	0.13
Male, *n* (%)	20 (77)	61 (73)	22 (76)	0.88	0.89
BSA, m^2^	1.65 ± 0.22	1.70 ± 0.21	1.68 ± 0.23	0.57	0.39
Systolic BP, mmHg	104 [90–120]	103 [90–120]	110 [100–124]	0.22	0.19
Diastolic BP, mmHg	65 [52–71]	63 [55–73]	68 [58–83]	0.09	0.09
Heart rate, bpm	80 [70–97]	75 [66–86]	90 [70–110]	< 0.01	< 0.01
HF duration, year	1.5 [0.1–6.8]	0.5 [0.0–6.8]	0.0 [0.0–1.3]	0.08	0.11
New-onset HF	6 (23)	28 (33)	16 (55)*^†^	0.03	0.04
Hemoglobin, mg/dL	13.5 ± 2.0	13.7 ± 2.0	14.3 ± 2.3	0.29	0.24
Sodium, meq/L	140 [136–141]	140 [138–141]	140 [138–142]	0.07	0.05
Creatinine, mg/dL	1.0 [0.8–1.6]	1.0 [0.8–1.2]	0.9 [0.8–1.1]	0.44	0.48
Total bilirubin, mg/dL	0.8 [0.6–1.4]	0.7 [0.5–0.9]	0.8 [0.6–1.3]	0.02	0.02
AST, U/L	27 [20–39]	25 [21–33]	28 [22–42]	0.16	0.16
ALT, U/L	26 [17–40]	25 [16–35]	35 [17–49]	0.08	0.08
BNP, ng/L	520 [285–1123]	392 [178–815]	682 [249–1338]	0.16	0.12
β-blockers	25 (96)	81 (96)	27 (93)	0.77	0.82
ACEI or/and ARBs	24 (92)	73 (87)	26 (90)	0.72	0.71
Diuretics	22 (85)	67 (80)	21 (72)	0.53	0.53
OMT score	4 [3–5]	4 [3–5]	5 [4–6]	0.19	0.21
Atrial fibrillation	5 (19)	8 (10)	3 (10)	0.43	0.39
RV pacing	3 (11)	5 (6)	0 (0)	0.09	0.21

ALT, alanine aminotransferase; AST, aspartate aminotransferase; BNP, brain natriuretic peptide; BSA, body surface area; BP, blood pressure; ACEI, angiotensin-converting enzyme inhibitor; ARB, angiotensin II receptor blocker; CRT, cardiac resynchronization therapy; HF, heart failure; OMT, optimal medical therapy; PM, pacemaker

Values are expressed as median [interquartile range] or *n* (%)

*p < 0.05, apical relatively-impaired group

^†^*p* < 0.05, scattered/homogeneously impaired group

**Table 2:** Echocardiographic data

Characteristic	Apical relatively-impaired group (*n* = 26)	Scattered/homogeneously impaired group (*n* = 84)	Apical relatively preserved group (*n* = 29)	*p* value	Adjsuted *p* value
LVDd, mm	67 [60–73]	67 [62–74]	68 [63–75]	0.98	0.98
LVDs, mm	62 ± 12.3	61 ± 11	63 ± 9	0.77	0.79
RVDd, mm	33 ± 8	36 ± 8	37 ± 8	0.36	0.41
LVEF, %	21 [18–28]	25 [20–30]	18 [16–27]^†^	< 0.01	< 0.01
LAVI, mL/m^2^	59 [36–77]	62 [43–80]	62 [53–85]	0.92	0.89
*E* wave, m/s	0.68 [0.51–0.93]	0.78 [0.53–0.90]	0.82 [0.55–0.97]	0.76	0.73
*e*′, ms/s	5.4 [4.0–7.0]	6.0 [4.2–7.0]	5.2 [3.5–7.1]	0.68	0.62
DT, ms	140 [104–175]	151 [113–187]	116 [91–156]^†^	0.02	0.02
*E*/*A*	1.4 [0.7–2.0]	1.5 [0.7–2.8]	1.6 [1.0–2.0]	0.57	0.59
*E*/*e*′	10.8 [7.8–15.6]	12.3 [9.3–16.5]	14.0 [10.4–20.0]	0.38	0.30
TAPSE, mm	11.0 [11.0–14.3]	16.0 [12.8–18.7]*	13.0 [11.0–14.0]^†^	< 0.01	< 0.01
TR-PG, mmHg	21.0 [15.0–30.0]	24.0 [18.0–32.0]	25.0 [18.0–30.0]	0.81	0.81
Systolic wall stress, × 10^3^ dynes/cm^2^	231 [167–345]	182 [139–251]	201 [152–281]	0.07	0.05
Diastolic wall stress, × 10^3^ dynes/cm^2^	47.9 [32.9–74.6]	49.5 [33.9–68.3]	50.1 [36.8–77.5]	0.70	0.76
GLS, %	− 4.6 [− 3.0–− 5.9]	− 7.4 [− 5.3 to − 9.1]*	− 4.8 [− 3.6 to − 6.7]^†^	< 0.01	< 0.01
RapLSI	0.14 [0.06–0.21]	0.46 [0.39–0.58]*	1.01 [0.84–1.18]*^†^	n.a.	n.a.
Average basal LS, %	− 5.9 [− 4.7 to − 8.0]	− 6.9 [− 5.3 to − 8.8]	− 3.1 [− 2.1 to − 4.1]*^†^	< 0.01	< 0.01
Average mid-LS, %	− 5.4 ± 3.4	− 7.6 ± 2.9*	− 4.4 ± 2.4^†^	< 0.01	< 0.01
Average apical LS, %	− 1.6 ± 1.5	− 7.0 ± 2.7*	− 7.6 ± 3.2*	< 0.01	< 0.01

DT, deceleration time; EF, ejection fraction; *E*, early diastolic transmitral flow velocity; *e*′, early diastolic mitral annular velocity; GLS, global longitudinal strain; LAVI, left atrial volume index; LS, longitudinal strain; LVDd, left ventricular end-diastolic diameter dimension; LVDs, left ventricular end-systolic diameter dimension; RVDd, right ventricular end-diastolic diameter dimension; RapLSI, relative apical longitudinal strain index; TAPSE, tricuspid annular plane systolic excursion; TR-PG, tricuspid regurgitation pressure gradient

Values are expressed as median [interquartile range] or *n* (%)

*p < 0.05, apical relatively-impaired group

^†^*p* < 0.05, scattered/homogeneously impaired group

### Relative apical longitudinal strain index and outcomes

**Fig. 2:**
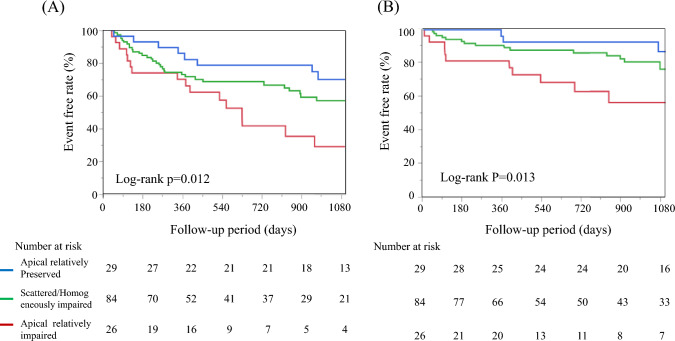
Event-free survival in patients with DCM and reduced EF, based on the RapLSI. The Kaplan–Meier plots of the event-free (A: cardiac death, hospitalization for HF, or LVAD implantation, B: cardiac death or LVAD implantation) survival in patients with HF and reduced EF who are stratified into the apical relatively preserved group (blue), scattered/homogeneously impaired group (green), and apical relatively-impaired group (red), calculated by the RapLSI. DCM, dilated cardiomyopathy; RapLSI, relative apical longitudinal strain index; LVAD, left ventricular assist device; HF, heart failure; and EF, ejection fraction

We observed the cardiac events, namely 13 cardiac deaths, 17 LVAD implantations, and 52 hospitalizations for HF during the follow-up (median [interquartile range]: 21 months [8–36]). The Kaplan–Meier survival analysis revealed that patients with different LS patterns had different cardiac event rates (Fig. 2). We performed univariate analyses with variables that were clinically important or different between groups (Table 3). The lower RapLSI, indicating an apical relatively-impaired pattern of the LS, institution, and new-onset HF were associated with a higher cardiac event rate, whereas the GLS, EF, and OMT scores were not in this study. In the multivariable analysis including new-onset HF, institution, OMT scores, and GLS, the association of RapLSI and LS distribution patterns with cardiac events remained significant (Table 4). The rate of LVRR was significantly higher in the apical relatively preserved group (52%) than in other groups (vs. apical relatively-impaired group (8%), *p* < 0.01; vs. scattered/homogeneously impaired group (25%), *p* < 0.01). After adjustment for new-onset HF, the association between achieving LVRR and LS distribution patterns remained significant (*p* < 0.01).

**Table 3:** Cox proportional-hazards regression analysis for the cardiac events

	HR	95% CI	*p* value
Age (per 1 year increase)	1.01	0.99–1.03	0.26
Male	0.92	0.50–1.69	0.79
Heart rate (per 1 bpm increase)	0.99	0.98–1.00	0.17
New-onset HF	0.45	0.24–0.82	< 0.01
Institution	2.94*	0.91–9.49	0.03
Beta-blockers use	0.77	0.23–2.34	0.63
ACEI/ARB use	0.69	0.31–1.53	0.39
OMT score (per 1 point increase)	1.07	0.88–1.32	0.47
Total bilirubin (1 mg/dL increase)	1.33	0.81–2.09	0.25
Log BNP (per 1.0 log unit increase)	1.09	0.86–1.38	0.49
LVEF (per 1% increase)	1.00	0.97–1.04	0.91
DT (per 1 ms increase)	1.00	0.99–1.00	0.26
TAPSE (per 1 mm increase)	1.05	0.96–1.15	0.26
GLS (per 1% increase)	1.00	0.92–1.11	0.85
RapLSI (per 0.1 decrease)	1.11	1.03–1.19	< 0.01
Average basal LS (per 1% increase)	0.96	0.90–1.05	0.33
Average mid-LS (per 1% increase)	1.00	0.92–1.09	1.00
Average apical LS (per 1% increase)	1.08	0.99–1.17	0.08
*Apical relatrviely preserved pattern*			
vs. scattered/homogeneously impaired pattern	0.57	0.26–1.23	0.15
vs. Apical more impaired pattern	0.30	0.13–0.71	< 0.01
*Apical relatively-impaired pattern*			
vs. scattered/homogeneously impaired pattern	1.88	1.03–3.43	0.04
vs. apical relatively preserved pattern	3.31	1.41–7.79	< 0.01

HR, hazard ratio; CI, confidence interval. Other abbreviations as in Tables 1 and 2

*HR of Osaka University Hopital to the University of Tsukuba Hospital

**Table 4:** Associations between RapLSI or LS ditribution patterns and risk of cardiac events

	RapLSI (per 0.1 decrease)		Apical relatively-impaired pattern			
			vs. scattered/homogeneously impaired pattern		vs. apical relatively preserved pattern	
	HR (95% CI)	*p* value	HR (95% CI)	*p* value	HR (95% CI)	*p* value
Model 1	1.08 (1.00–1.17)	0.04	1.66 (0.89–3.08)	0.11	2.48 (1.00–6.18)	0.049
Model 2	1.11 (1.03–1.19)	< 0.01	1.81 (0.94–3.48)	0.08	3.20 (1.31–7.83)	0.01
Model 3	1.16 (1.06–1.27)	< 0.01	1.89 (1.02–3.49)	0.04	3.50 (1.41–8.67)	< 0.01
Model 4	1.11 (1.03–1.19)	< 0.01	1.84 (0.99–3.41)	0.05	3.18 (1.30–7.80)	0.01
Model 5	1.10 (1.03–1.18)	< 0.01	1.77 (0.96–3.28)	0.07	3.26 (1.33–8.00)	< 0.01

Abbreviations as in Tables 1, 2, and 3

Model 1, adjusted for age, sex, and new-onset HF; model 2, adjusted for age, sex, and GLS; model 3, adjusted for age, sex, and institution; model 4, adjusted for age, sex, and beta-blocker; model 5, adjusted for age, sex, and OMT score

## Discussion

Our study reported two major findings. First, we showed that severe impairment in LS in the LV was not always homogeneous or uniform in patients with reduced EF. Second, we showed that the regional pattern, expressed by RapLSI, was associated with outcomes. Patients with an apical relatively-impaired pattern had a higher cardiac event rate (cardiac death, LVAD implantation, or HF hospitalization) and achieved LVRR less frequently than those with an apical more preserved pattern. In this population, EF and GLS were not associated with outcomes. RapLSI was not correlated with EF and GLS. These results indicate that RapLSI may have different clinical information from EF and GLS. Evaluating the LS distribution pattern would facilitate risk stratification in DCM patients with reduced LVEF.

### Patient characteristics in the apical relatively preserved group

No studies reported the distribution patterns of LS in DCM patients. Kusunose K et al. reported that the patients with tachycardia-induced cardiomyopathy had lower RapLSI values [13]. However, the RapLSI values and GLS appeared to be different from those in this study and mostly within the range of the scatter/homogeneously impaired group. Despite vendor differences in evaluating LS between the two studies, this may relate to the different outcomes in the “apical relatively preserved group.” In this study, clinical features of the apical more preserved group were a short duration of HF and better outcomes including a high occurrence of LVRR compared with those in other groups. Although not all LS data at the 1-year follow-up were evaluated, decreased GLS improved in patients who had achieved LVRR after a 1-year follow-up. The RapLSI values became close to 0.5, indicating a change in the LS pattern from an apical relatively preserved to a scattered or homogeneously impaired pattern (Supplemental Fig. 1). On the other hand, GLS and LS patterns did not change in patients without LVRR (Figure S1). These data indicate that LS in the apical region may be preserved in the early stages of DCM. In addition, the basal myocardium may be vulnerable to stress, and a temporal decrease in LS may be attributed to the increased wall stress [19].

### Patient characteristics of the apical relatively-impaired pattern

Patients with an apical relatively-impaired pattern had worse outcomes than those with an apical relatively preserved pattern. The HF duration was weakly correlated with apical LS values. Shorter HF duration or disease history was associated with LVRR and prognosis [4, 20]. Based on our results regarding the associations of LS impaired pattern or GLS values with the HF duration or the LVRR, we hypothesized that advanced DCM is characterized by a severe decrease in LS in the apex. This may be related to morphologic changes in the apex due to advanced remodeling toward spheroidization of the LV. Alternatively, differences in the amount of myocardium, distribution of beta-adrenergic receptors [21], or hemodynamic load may lead to varying progression of myocardial injury in the apex. As the progression of GLS impairment is considered to be disease progression, the pattern of LS and GLS may reflect the disease trajectory of DCM (Supplemental Fig. 2). Further studies are needed to confirm our hypothesis because we did not show enough serial data of GLS and LS patterns in study patients. Patients with an apical relatively-impaired pattern tended to more frequently have an RV-paced rhythm. This may be due to myocardial damage that required pacing because a paced rhythm was associated with higher cardiac event rates. In our preliminary assessment, RapLSI at turning on and off the pacing did not much differ (data not shown). Despite excluding patients with pacing from the analysis, RapLSI was significantly associated with cardiac events. Therefore, pacing exerts a limited effect on the RapLSI values.

### Limitations

Our study had several limitations. We conducted a retrospective study with a small sample size, and sample numbers at each institution were not even. However, we believe that the results provided a new insight for assessing disease progression that is worth testing on a larger scale. We excluded patients with poor echo views. Various kinds of vendors used for LS acquirements were used, but they were not different among the three groups. We used one analyzing software (TOMTEC) to reduce the difference by vendors, but the strain values may vary according to vendor differences, making it difficult to compare the absolute values to those of other studies. Errors in RapLSI will be greater in patients with lower GLS. The impact of the absolute difference in LS values between the apex and the other segments becomes greater at the lower GLS. However, RapLSI was associated with clinical outcomes after adjustment for GLS. Our findings should be confirmed with other imaging modalities or prospective studies using serial LS data. We used BNP levels on admission, which were not measured at the same point of echocardiography.

## Conclusion

DCM patients with different distributions of impaired LS had different outcomes. The LS pattern may reflect disease progression and enable risk stratification in DCM with reduced LVEF.

## Supplementary Information

Below is the link to the electronic supplementary material.

Supplementary file1 (DOCX 429 KB)

## Data Availability

The datasets generated and/or analyzed during the current study will be partial available from the corresponding author upon reasonable request.

## Abbreviations

BNP: Brain natriuretic peptide
DCM: Dilated cardiomyopathy
EF: Ejection fraction
GLS: Global longitudinal strain
HF: Heart failure
LS: Longitudinal strain
LV: Left ventricular
LVAD: Left ventricular assist device
LVRR: Left ventricular reverse remodeling
OMT: Optimal medical therapy
RapLSI: Relative apical longitudinal strain index
RV: Right ventricular
