# Supplementary material for: Diverse distribution patterns of segmental longitudinal strain are associated with different clinical features and outcomes in dilated cardiomyopathy
Source: J Echocardiogr. 2024 Mar 7;22(4):193–201. doi: 10.1007/s12574-024-00646-y (PMC11561091; doi:10.1007/s12574-024-00646-y)
Supplement: Supplementary file 1 — Supplementary file1 (DOCX 429 KB) [file 12574_2024_646_MOESM1_ESM.docx]

**SUPPLEMENTAL MATERIAL**

**Supplemental Figure 1.**

**
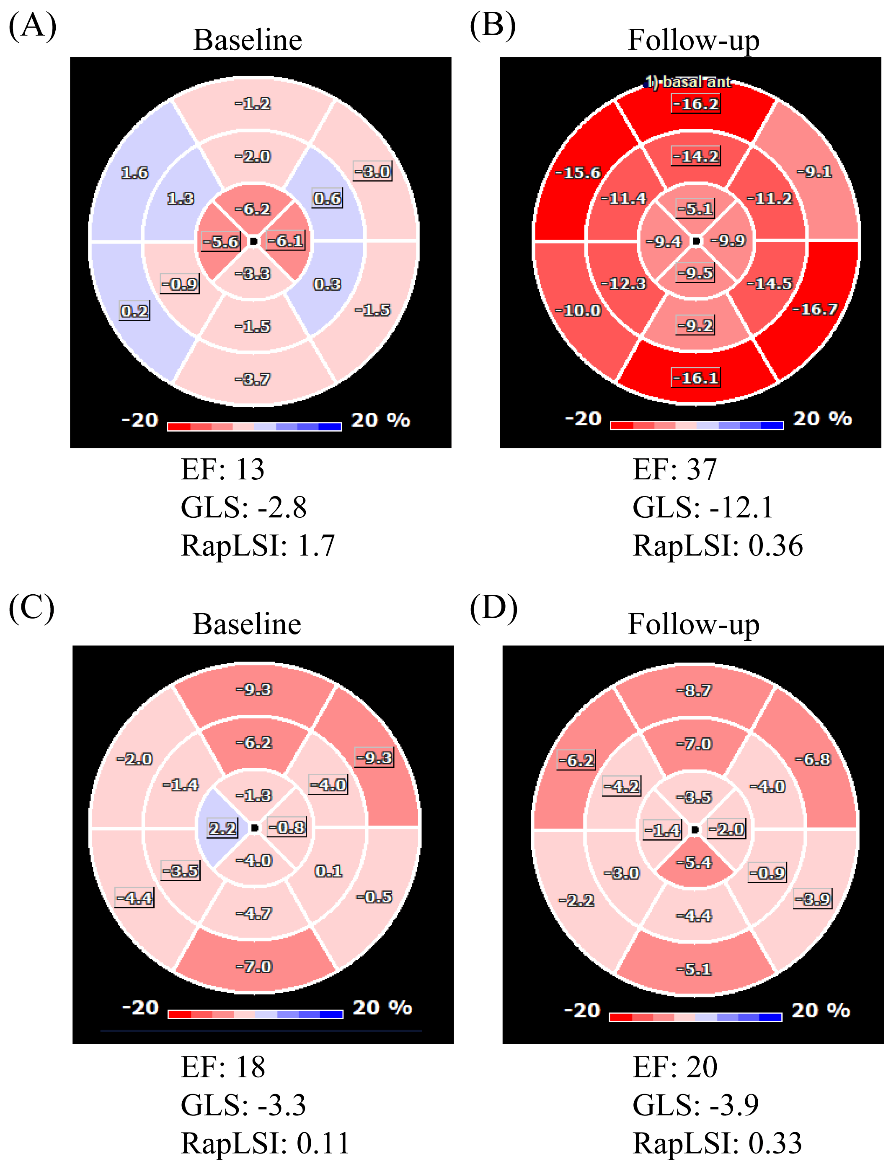
**

Presentative cases of serial changes in the LS pattern and GLS in patients with DCM and LVRR (A and B), and without LVRR (C and D).

LS, longitudinal strain; GLS, global longitudinal strain; DCM, dilated cardiomyopathy; and LVRR, left ventricular reverse remodeling.

**Supplemental Figure 2.**


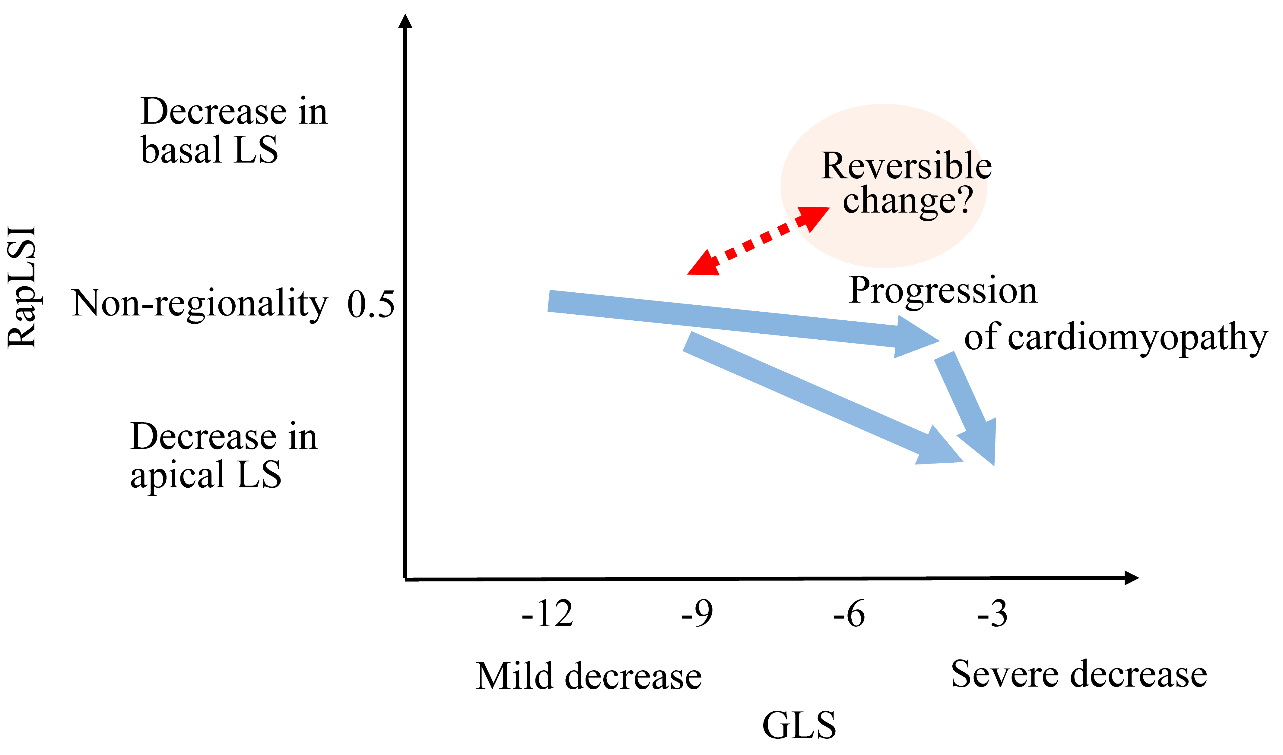


The working hypothesis of GLS trajectories and LS distribution patterns, represented by RapLSI, in the development of DCM.

LS, longitudinal strain; GLS, global longitudinal strain; DCM, dilated cardiomyopathy; and RapLSI, relative apical longitudinal strain index.

**Supplemental Table 1. Patient characteristics in the two institutions**

| **Characteristic** | **Osaka Univesity Hospital**  **(n=114)** | **The University of Tsukuba Hospital**  **(n=25)** | **p-value** |
| --- | --- | --- | --- |
| Age, years | 54 [42-66] | 59 [47-68] | 0.42 |
| Male sex | 85 (75) | 18 (72) | 0.79 |
| BSA, m² | 1.68 ± 0.20 | 1.72 ± 0.28 | 0.38 |
| Systolic BP, mmHg | 104 [90-120] | 112 [98-123] | 0.55 |
| Diastolic BP, mmHg | 65 [54-73] | 62 [56-75] | 0.78 |
| Heart rate, bpm | 80 [68-94] | 76 [64-93] | 0.44 |
| HF duration, year | 0.5 [0.0-4.0] | 1.0 [0.0-9.5] | 0.07 |
| New-onset HF | 43 (38) | 7 (28) | 0.35 |
| Hemoglobin, mg/dL | 13.7 ± 2.1 | 14.2 ± 1.9 | 0.28 |
| Sodium, meq/L | 140 [137-141] | 140 [139-142] | 0.09 |
| Creatinine, mg/dL | 0.9 [0.8-1.2] | 1.0 [0.8-1.2] | 0.50 |
| Total bilirubin, mg/dL | 0.8 [0.5-1.0] | 0.8 [0.6-1.2] | 0.69 |
| AST, U/L | 27 [21-36] | 25 [18-36] | 0.98 |
| ALT, U/L | 26 [17-40] | 23 [14-40] | 0.74 |
| BNP, ng/L | 419 [207 - 878] | 517 [243 - 1154] | 0.15 |
| β-blockers | 108 (95) | 25 (100) | 0.11 |
| ACEI or/and ARBs | 108 (95) | 15 (60) | <0.01 |
| Diuretics | 90 (79) | 20 (80) | 0.91 |
| OMT score | 4[4-5] | 4[3-5] | 0.50 |
| Atrial fibrillation | 14 (12) | 2 (8) | 0.53 |
| RV pacing | 5 (4) | 3 (12) | 0.17 |

Abbreviations as in Table 1
Values are expressed as median [interquartile range] or n (%).

**Supplemental Table 2. Echocardiographic data in the two institutions**

| **Characteristic** | **Osaka Univesity**  **Hospital**  **(n=114)** | **The University of Tsukuba Hospital**  **(n=25)** | **p-value** |
| --- | --- | --- | --- |
| LVDd, mm | 67[61-76] | 67 [64-73] | 0.68 |
| LVDs, mm | 62 ± 11.1 | 61 ± 8.0 | 0.68 |
| RVDd, mm | 37 ± 8 | 33 ± 8 | 0.03 |
| LVEF, % | 23 [18-29] | 22 [18-28] | 0.54 |
| LAVI, mL/m² | 64 [43-84] | 58 [47-75] | 0.33 |
| E wave, m/s | 0.73 [0.52-0.92] | 0.77 [0.60-1.06] | 0.44 |
| e’, ms/s | 6.0 [4.2-7.1] | 5.0 [4.0-6.4] | 0.18 |
| DT, ms | 139 [107-183] | 145 [121-188] | 0.73 |
| E/A | 1.5 [0.8-2.2] | 1.7 [0.7-3.2] | 0.67 |
| E/e’ | 11.8 [8.8-16.6] | 14.1 [10.9-21.6] | 0.10 |
| TAPSE, mm | 14.0 [11.0-17.8] | 14.2 [11.7-18.7] | 0.39 |
| TR-PG, mmHg | 24.0 [18.0-30.0] | 25.0 [18.0-38.8] | 0.22 |
| Systolic wall stress, ×10³ dynes/cm² | 223 [147-293] | 163 [123-172] | <0.01 |
| Diastolic wall stress, ×10³ dynes/cm² | 50.2 [36.1-72.7] | 40.7 [31.2-70.3] | 0.15 |
| GLS, % | -6.7 [-4.7- -8.6] | -5.7 [-3.9- -7.2] | 0.07 |
| RapLSI | 0.48 [0.33-0.72] | 0.42 [0.29-0.54] | 0.01 |
| Average basal LS, % | -6.2 [-4.0- -8.5] | -4.9 [-3.6- -6.3] | 0.08 |
| Average mid LS, % | -6.6 ± 3.1 | -6.0 ± 3.5 | 0.40 |
| Average apical LS, % | -6.4 ± 3.4 | -4.8 ± 3.3 | 0.03 |

Abbreviations as in Table 2

Values are expressed as median [interquartile range] or n (%).
